# Prevalence of overweight and obesity on the island of Ireland: results from the North South Survey of Children's Height, Weight and Body Mass Index, 2002

**DOI:** 10.1186/1471-2458-7-187

**Published:** 2007-07-31

**Authors:** Helen Whelton, Janas Harrington, Evelyn Crowley, Virginia Kelleher, Michael Cronin, Ivan J Perry

**Affiliations:** 1 WHO Collaborating Centre for Oral Health Services Research, University College Cork, Ireland; 2 Department of Epidemiology and Public Health, University College Cork, Ireland; 3 Department of Statistics, University College Cork, Ireland

## Abstract

**Background:**

Childhood obesity is emerging as a major public health problem in developed and developing countries worldwide. The aim of this survey was to establish baseline data on the prevalence and correlates of overweight and obesity among children and adolescents in the Republic of Ireland (RoI) and Northern Ireland (NI).

**Methods:**

The heights and weights of 19,617 school-going children and adolescents aged between 4 and 16 years in NI and RoI were measured using standardised and calibrated scales and measures. The participants were a representative cross-sectional sample of children randomly selected on the basis of age, gender and geographical location of the school attended. Overweight and obesity were classified according to standard IOTF criteria.

**Results:**

Males were taller than females, children in RoI were taller than those in NI and the more affluent were taller than the less well off. The overall prevalence of overweight and obesity was higher among females than males in both jurisdictions. Overall, almost one in four boys (23% RoI and NI) and over one in four girls (28% RoI, 25% NI) were either overweight or obese. In RoI, the highest prevalence of overweight was among 13 year old girls (32%) and obesity among 7 year old girls (11%). In NI the highest prevalence of overweight and obesity were found among 11 and 8 year old girls respectively (33% and 13%).

**Conclusion:**

These figures confirm the emergence of the obesity epidemic among children in Ireland, a wealthy country with the European Union. The results serve to underpin the urgency of implementing broad intersectoral measures to reduce calorie intake and increase levels of physical activity, particularly among children.

## Background

Obesity is one of the major public health challenges of our time. In the US at least one quarter of the adult population is obese. In Ireland current estimates suggest that one in five adults is obese posing a particular threat to the population given our high mortality from cardiovascular disease relative to other European countries [1,2]. Over the past decade there is evidence of an emerging epidemic of childhood obesity worldwide [3,4] and it is suggested that children in established market economies, born at the start of the 21^st ^century, may have a shorter life expectancy than their parents as a result of the health consequences of obesity.

Clearly, obesity, particularly amongst children is a serious public health problem, however in order to affect appropriate public health measures accurate estimates of its prevalence are required. Previous surveys indicate that overweight and obesity are common in Ireland. Ireland ranked amongst the countries with the highest levels of obesity in a survey of self reported height and weight among 13 and 15 year olds in 13 European countries, Israel and the United States in 1997–1998 [5]. The highest prevalence of overweight was found in the United States, Ireland, Greece and Portugal. Data from the UK shows that an estimated 10% of 6-year-olds and 17% of 15-year-olds are obese [6]. Such is the concern about obesity in Ireland that the Minister for Health and Children appointed a National Task force to review the obesity trends in Ireland and make health promoting policy recommendations designed to address unfavourable trends. Establishment of baseline data with regard to overweight and obesity among Irish children and subsequent monitoring of these trends is important for the development of appropriate health policy and for the subsequent monitoring of the outcome of any interventions designed to tackle or prevent childhood overweight or obesity. The aim of this survey was to establish baseline data on the prevalence and correlates of overweight and obesity among children and adolescents in Ireland. This study was part of a larger national study of children's oral health [7].

This paper presents obesity prevalence rates from a North South Survey of Children's Height, Weight and Body Mass Index conducted in Ireland in 2001/'02. The study provides a baseline measurement of children's height and weight against which future change can be measured. By comparing these data with international norms we can estimate the current prevalence of overweight and obesity among children and adolescents in Ireland. The importance of appropriate information to the planning and evaluation of measures to deal with overweight and obesity in Ireland is clear. The results of this study will establish the extent of the obesity problem among children and adolescents in Ireland.

## Methods

### Ethical approval

The Ethics Committee of the Cork Teaching Hospitals reviewed the protocols for training and calibration of the examiners and for the main study. The committee approved the study on 2^nd ^October 2001. Ethical Approval was also obtained from the Research Ethics Committee of Queen's University, Belfast for the Northern part of the study.

### Sample

The primary sampling unit was the school. A cluster sampling technique was used with schools as the clustering unit. Schools were categorised according to, health board region and size (to ensure representation of schools of various sizes) and whether they were located in a fluoridated or non-fluoridated areas. Within each Community Care Area, schools were randomly selected to ensure a balance for fluoridation status (where appropriate) and proportionally to the size of the school. A list of children in each class in each year (Junior Infants-5 year olds-, 2^nd ^class-8 year olds-, 6^th ^class-12 year olds- and Junior Cert-15 year olds) was obtained from the selected schools.

Children were selected randomly on the basis of age, gender, and geographical location of the school attended and whether they attended a school with fluoridated or non-fluoridated water supply (necessary for the oral health survey). The age groups for inclusion in the survey were chosen to allow comparison of oral health data with earlier Irish studies and with studies conducted internationally. The groups chosen were children in Junior Infants, Second Class, Sixth Class and Junior Certificate in RoI and Primary 1, Primary 4, Year 1 and Year 4 in NI. Children in Junior Infants/Primary 1 are on average five years old, however these classes also have four and six year olds. Children in 2nd Class/Primary 4 are seven, eight and nine years old with eight year olds being the most common. In sixth class/Year 1 children are on average 12 years old but the class also has 11 and 13 year olds. Similarly in Junior Certificate/Year 4, adolescents are 14, 15 and 16 years old.

All children within a class were included in the random selection irrespective of whether they had special needs, but teams were told not to include in the selection whole classes that were designated as special needs within a school. Schools designated 'special needs' by the Department of Education and Science were the subject of a separate survey of oral health conducted in 2003 (report in preparation). The required number of children was selected randomly from each year and the consent forms were issued to only those children.

### Measurement of height and weight

Weights were measured using a Soehnle 7403 Mediscale. The weighing scales were calibrated using 75 kg calibration weights either in Cork by the OHSRC or by each team in their own Health Board prior to the commencement of the study. The scales were checked again at the end of the fieldwork. Leicester Height Measures, (CMS Weighting Equipment, 18 Camden High Street, London) were used to measure height. The protocol for measuring height and weight is detailed elsewhere [7].

### Data management and analyses

The data were recorded electronically and were processed and coded prior to analysis at the Oral Health Services Research Centre. The SAS statistical package was used for analysis.

### Definition of overweight and obesity

The Body Mass Index is an accepted method [8,9] for measuring childhood obesity. For adults a BMI of 25–30 kg/m^2 ^is the accepted definition of overweight (increased body weight when compared to established standards) and a BMI of >30 kg/m^2 ^is classified as obese (an abnormal accumulation of fat compared to established standards) The situation for children is more complex as weight changes with height and hence different cut-off points have to be defined for children at different ages. There are limited population norms for BMI for children in Ireland. There are no generally agreed BMI criteria for classifying overweight and obesity in children, however, there is an emerging consensus in favour of adopting criteria proposed by the International Obesity Task Force (IOTF) [10]. The latter criteria are used in this research.

### Other variables

Eligibility for state funded general medical and dental services (Medical card ownership) by the parents or guardians of the children and adolescents in the sample was used as a surrogate for disadvantage in RoI. Parents were asked to indicate whether they had a Medical Card, on the consent form, which was returned to the school prior to the clinical examination. For the general population under age 70 in RoI, Medical Card issue is based on a means test unless the applicant has a disability. Medical Cards are issued to low-income applicants. In NI disadvantage was classified according to whether the parents or guardians of the children or adolescents in the sample were in receipt of any Low-Income Benefits.

## Results

### Response rate

The overall response rate for RoI was 68% (68%, 68%, 68% and 66% in the 5-, 8-, 12- and 15-year-old age groups, respectively). The overall response rate for NI was 53% (56%, 63%, 59% and 43%, respectively).

The number of children examined and the distribution of the sample by age and gender, for the Republic of Ireland and Northern Ireland are presented in table 1. Although, data are available for a wide age range, the modal ages examined were 5, 8, 12 and 15 year olds. In RoI the numbers examined were large enough to provide data at national level for the age groups at either side of the modal age (except for 10 year olds). For the NI sample, data are presented for ages 4, 5, 8, 11, 12, 14 and 15 where there were at least 47 children in each age/gender group. The gender distribution was balanced in RoI, slightly more females than males were examined in NI (51% vs 49%). The mean age of the children was comparable allowing meaningful comparisons of RoI and NI data.

**Table 1:** Number of children examined (with height and weight recorded) by class and gender in the Republic of Ireland and Northern Ireland

RoI			NI		
CLASS	Male (N)	Female (N)	CLASS	Male (N)	Female (N)

Primary Year 1	3327	3151	Primary One	408	419
Primary Year 4	1872	1867	Primary Four	158	144
Primary Year 6	1859	1968	Year One	162	181
Second Level Year 3	1748	1726	Year Four	307	320
TOTAL	8806	8712	TOTAL	1035	1064

### Body mass index (BMI)

Details of height and weight distribution have been reported elsewhere [7]. In summary however, the results showed that males were taller than females, children in the Republic of Ireland were taller than those in NI and the less well off were smaller than the rest of the population. Height is a fundamental indicator of growth and development with well documented secular trends linked to nutrient intakes and the markers of socioeconomic status. There was evidence that socio economic disadvantage was associated with shorter stature across the age ranges for both jurisdictions. The pattern for weight differed to that for height. The increase in weight was less linear and followed a more S shaped curve, with periods of greater weight gain between age 6–8 and 9–12 for both sexes. Boys in NI tended to be lighter than boys in RoI until age 14 when they were slightly heavier. By age 15 the weights were the same. Girls in NI were lighter at age 4, the same weight at ages 5 and 8, slightly lighter at age 11 and 12, lighter at age 14 (3.2 kg) and the same weight at age 15 years.

The mean BMI by age and gender is shown in table 2 for both RoI and NI. Among RoI and NI males BMI decreased by 0.1 kg/m^2 ^between age 4 (16.7 kg/m^2) ^and 5 (16.6 kg/m^2) ^as weight increased faster than height. In RoI males, BMI started to increase from age 5 to age 16 (21.8 kg/m^2^). For females in RoI, weight increased at the same rate as height from age 4–6 after which weight gain surpassed height increase and the mean BMI increased with age from 16.6 kg/m^2 ^at age six to 22.5 kg/m^2 ^at age 16. Among NI girls, BMI increased from 16.1 kg/m^2 ^to 16.6 kg/m^2 ^between age 4 and 5 and from 16.6 at age 5 to 22.2 kg/m^2 ^at age 15. There was a tendency for girls to have higher BMI levels than boys (Table 2) and the increase in mean BMI with age is evident. Comparing RoI and NI, there were no consistent differences in BMI by age and gender according to jurisdiction.

**Table 2:** BMI Mean and standard deviation by age and gender for the Republic of Ireland and Northern Ireland

Republic of Ireland						
	Male			Female		

Age (Yrs)	N	Mean (Std)	95% CI	N	Mean (Std)	95% CI

4	630	16.7 (1.6)	16.6–16.8	772	16.6 (1.9)	16.5–16.7
5	2474	16.6 (1.6)	16.5–16.7	2278	16.6 (2)	16.5–16.7
6	224	16.8 (2.1)	16.5–17.0	152	16.6 (1.9)	16.3–16.9
7	338	17.1 (2.2)	16.9–17.4	451	17.6 (3)	17.3–17.9
8	1372	17.6 (2.8)	17.5–17.8	1327	17.9 (2.9)	17.7–18.1
9	152	17.9 (3.3)	17.4–18.4	85	18.2 (3.6)	17.4–19
11	327	19.5 (3.4)	19.2–19.9	438	20.3 (3.8)	19.9–20.7
12	1325	19.9 (3.6)	19.7–20.1	1381	20.3 (4)	20.1–20.5
13	206	20.1 (3.5)	19.6–20.6	154	20.9 (3.9)	20.2–21.5
14	608	21.2 (3.7)	20.9–21.5	708	22.1 (3.8)	21.9–22.4
15	1051	21.7 (3.5)	21.5–21.9	935	22.2 (3.8)	21.9–22.4
16	88	21.8 (3.4)	21.1–22.5	73	22.5 (4.4)	21.4–23.5

Northern Ireland						

	Male			Female		

Age (Yrs)	N	Mean (Std)	95% CI	N	Mean (Std)	95% CI

4	50	16.7 (1.4)	16.3–17.1	54	16.2 (1.2)	15.9–16.5
5	356	16.6 (1.7)	16.4–16.8	365	16.6 (1.9)	16.4–16.8
8	131	17.1 (1.9)	16.8–17.4	119	18.1 (3.3)	17.5–18.7
11	47	19.3 (2.9)	18.4–20.1	57	20.3 (3.8)	19.3–21.4
12	115	19.7 (3.3)	19.1–20.3	122	20.2 (3.8)	19.5–20.8
13						
14	108	21.6 (3.8)	20.9–22.4	86	21.1 (3)	20.4–21.7
15	198	21.7 (3.6)	21.2–22.2	231	22.2 (3.6)	21.7–22.6

An analysis of variance was carried out to examine the impact of gender (M/F), jurisdiction (RoI/NI), Medical Card or Low Income Benefit status of parents (yes/no) and Age (to nearest year) on BMI (after natural log transformation).

There was a significant difference between males and females (p < 0.0001), with females having higher mean BMI. There was also a significant difference between the disadvantaged and others (p = 0.0062). The less well off, had a higher mean BMI. There was a significant effect for age, with BMI increasing with age (p < 0.0001). The increase in BMI with age was greater for females than males (p < 0.0001) and for medical card holders than non-medical card holders (p = 0.0008). There was no difference in mean BMI between RoI and NI (p = 0.1317).

### Overweight and obesity

#### Prevalence of overweight and obesity according to age and gender in RoI and NI

The prevalence of overweight and obesity according to IOTF classification are presented by age and gender for RoI and NI, in Table 3. Overall, almost one in four boys (23% RoI and NI) and over one in four girls (28% RoI, 25% NI) were either overweight or obese. About one in 20 boys (6% in RoI, 5% in NI) and about one in 15 girls (7% in RoI and NI) aged 2–16 were obese in 2002, according to the International Classification. The overall prevalence of overweight was higher among females than males in the RoI (28% vs 23%) and NI (25% vs 23%). Similarly, the prevalence of obesity was higher in girls in both jurisdictions (RoI 7%vs.6% and NI 7% vs. 5%).

**Table 3:** Percentage of children overweight and obese using IOTF criteria by age and gender in the Republic of Ireland and Northern Ireland

Republic of Ireland						
	Male			Female		

Age (Yrs)	N	% Overweight (incl. obese)	% Obese	N	% Overweight (incl. obese)	% Obese

4	630	26	7	722	29	7
5	2474	22	5	2278	29	7
6	224	18	5	152	29	7
7	338	20	7	451	30	11
8	1372	24	7	1327	30	8
9	152	22	9	85	31	9
11	327	27	6	438	29	8
12	1325	25	6	1381	25	6
13	206	20	4	154	32	5
14	608	22	6	708	27	7
15	1051	22	5	935	22	5
16	88	20	5	73	21	5
ROI	8795	23	6	8704	28	7

Northern Ireland						

	Male			Female		

Age (Yrs)	N	% Overweight (incl. obese)	% Obese	N	% Overweight (incl. obese)	% Obese

4	50	28	4	54	22	0
5	356	22	5	365	28	7
8	131	18	1	119	23	13
11	47	23	4	57	33	9
12	115	23	6	122	27	7
14	108	26	7	86	14	1
15	198	25	6	231	23	6
NI	1005	23	5	1034	25	7

Across age groups, overweight was most common among 13 year old girls (32%) and obesity most common in 7 year old girls (11%) in the RoI. In NI the highest prevalence of overweight and obesity were found among 11 and 8 year old girls respectively (33% and 13%).

#### Prevalence of overweight and obesity according to disadvantage in RoI and NI

**Figure 1:**
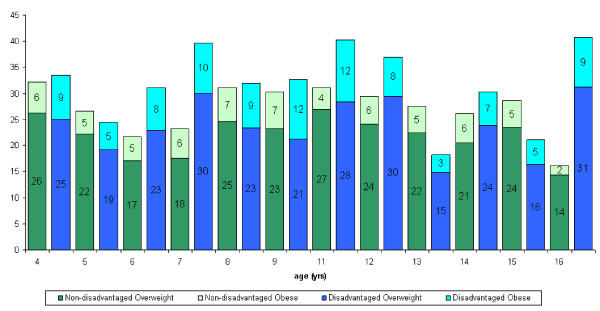
Distribution of overweight and obese females in the RoI according to disadvantage and age.

**Figure 2:**
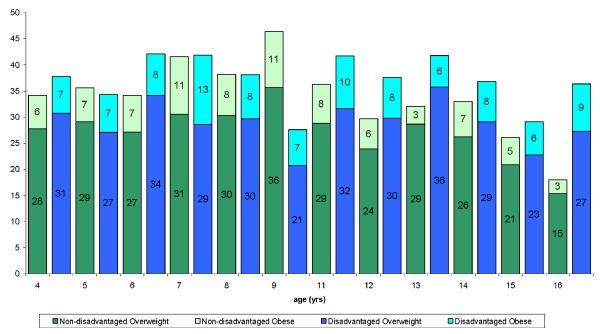
Distribution of overweight and obese males in the RoI according to disadvantage and age.

**Figure 3:**
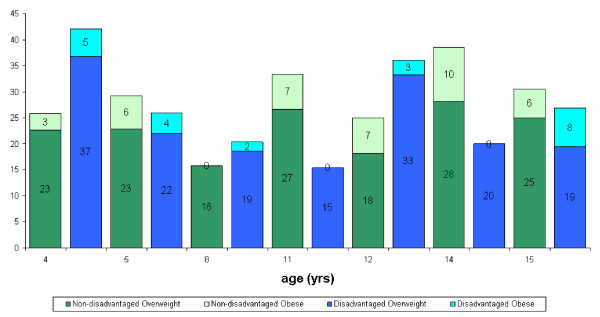
Distribution of overweight and obese females in NI according to disadvantage and age.

**Figure 4:**
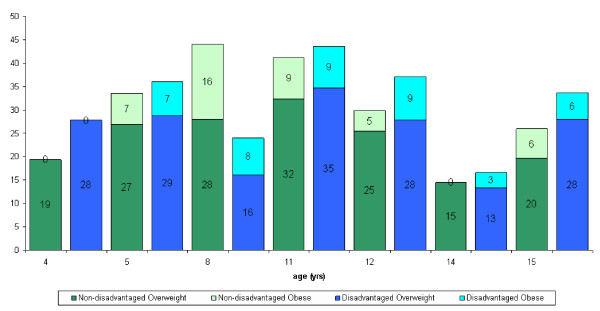
Distribution of overweight and obese males in NI according to disadvantage and age.

Twenty four per cent of the total sample examined in RoI were dependants of parents with medical cards. In NI 38% of the sample were from families in receipt of low-income benefits. The data were analysed according to the occupational status of the parents. No consistent trends were observed in either RoI or NI (figure 1, 2, 3, 4).

## Discussion

This survey presents data on the height, weight and body mass index of a nationally representative sample of children aged 4–16 years in the Republic of Ireland and Northern Ireland. Using international norms, overall, almost one in four boys (23% RoI and NI) and over one in four girls (28% RoI, 25% NI) were either overweight or obese. While not significant, higher rates of overweight and obesity were seen in the Republic of Ireland compared to Northern Ireland.

Current data in Ireland from the recent National Health and Lifestyle Survey, SLÁN indicate that between 1998 and 2002 obesity rates in adults rose by 3% and Health Behaviours in School Children (HBSC) figures indicate that 14% of 13 year old boys and 10% of 13 year old girls are either overweight or obese [11]. Both of these studies are based upon self reported heights and weights. To date however, no data were available to allow a North-South comparison in Ireland. The pattern of overweight and obesity found in this survey (2002) is very similar to that observed in the Health Survey for England 2002 [12]. In the latter survey, according to IOTF criteria, 6% of boys and 7% of girls aged 2–15 were obese and 22% of boys and 28% of girls were either overweight or obese. In Ireland in 2002, almost one in four boys (23% RoI and NI) and over one in four girls (28% RoI and 25% NI) were either overweight of obese. Had the UK 1990 cut-off points of the 91^st ^and 98^th ^centile, for overweight and obesity respectively, these figures are substantially higher.

A novel aspect of the research is that it is the first all Ireland study in which the height and weight of a representative sample of children has been measured contemporaneously, north and south of the border, using standardised criteria. The study, is the largest of it's kind estimating the prevalence of overweight and obesity amongst school-aged children in Ireland to date (n = 19,617). There was a good response rate particularly for the Republic of Ireland, 68% and 53% Northern Ireland, allowing confidence that the estimated prevalence rates are reliable. However, surprisingly, there was no significant variation in prevalence across social groups in either RoI or NI. The measure of socioeconomic status recorded for this study was the possession of a General Medical Services (GMS) Card in RoI and those in receipt of Low Income Benefits (LIB) in NI. It is probable that a more significant variation in prevalence of obesity among school-aged children across the island of Ireland would be seen with a more complete measure of socio-economic status. Additionally, the difference in the response rate between RoI and NI requires us to interpret the North-South difference with caution.

The extent to which non response bias has affected these results is unclear. However, one could assume that if subjects were likely to refuse consent for the study on the basis of weight, they would probably be overweight subjects. This would result in an underestimate of overweight and obesity. The fact that this was primarily a dental survey and that assurances of privacy and confidentiality were given in the consent form is likely to have minimised non response due to embarrassment as a result of overweight. The same challenges are faced by other similar studies and these challenges are difficult to overcome.

## Conclusion

In conclusion, this study provides further compelling evidence on the emergence of the obesity epidemic among children in Ireland. Given the burden of disease linked with obesity, in particular Type II Diabetes Mellitus, these findings have significant implications for population health and health care costs over the coming decades.

## Competing interests

The author(s) declare that they have no competing interests.

## Authors' contributions

HW conceived of the study, and participated in the design and coordination and helped to draft the manuscript. JH helped to draft the manuscript. EC calibrated the fieldworkers and the scales. VK processed the data and produced the tables. MC performed the statistical analysis. IP participated in the design of the study and helped draft the manuscript. All authors read and approved the final manuscript.

## Pre-publication history

The pre-publication history for this paper can be accessed here:


